# Potential Use of Tomato Peel, a Rich Source of Lycopene, for Cancer Treatment

**DOI:** 10.3390/molecules29133079

**Published:** 2024-06-28

**Authors:** Diana Carolina Jiménez Bolaño, Daniel Insuasty, Juan David Rodríguez Macías, Carlos David Grande-Tovar

**Affiliations:** 1 Grupo de Investigación de Fotoquímica y Fotobiología, Universidad del Atlántico, Carrera 30 Número 8-49, Puerto Colombia 081008, Colombia; dcarolinajimenez@mail.uniatlantico.edu.co; 2 Departamento de Química y Biología, División de Ciencias Básicas, Universidad del Norte, Km 5 Vía Puerto Colombia, Barranquilla 081007, Colombia; insuastyd@uninorte.edu.co; 3 Programa de Medicina, Facultad de Ciencias de la Salud, Universidad Libre, Km 5 Vía Puerto Colombia, Barranquilla 081007, Colombia; juand.rodriguezm@unilibre.edu.co

**Keywords:** antioxidant, cancer therapy, lycopene, tomato peel, waste re-use

## Abstract

Tomatoes are well known for their impressive nutritional value among vegetables. However, the industrial processing of tomatoes generates a significant amount of waste. Specifically, 10% to 18% of the raw materials used in tomato processing become waste. This waste can seriously affect ecosystems, such as freshwater bodies, wetlands, rivers, and other natural environments, if not properly managed. Interestingly, tomato waste, specifically the skin, contains lycopene, a potent antioxidant and antimutagenic that offers a range of health benefits. This makes it a valuable ingredient in industries such as food and cosmetics. In addition, researchers are exploring the potential of lycopene in the treatment of various types of cancer. This systematic review, guided by the PRISMA 2020 methodology, examined studies exploring the possibility of tomato peel as a source of lycopene and carotenoids for cancer treatment. The findings suggest that tomato peel extracts exhibit promising anticancer properties, underscoring the need for further investigation of possible therapeutic applications. The compiled literature reveals significant potential for using tomato peel to create new cancer treatments, which could potentially revolutionize the field of oncology. This underscores the importance of continued research and exploration, emphasizing the urgency and importance of the scientific community’s contribution to this promising area of study.

## 1. Introduction

The life cycle of food, from harvesting to industrial processing, generates considerable waste. This problem in agricultural production, industrial processing, and the distribution stage represents a major environmental and economic challenge [1]. Worldwide, managing food waste represents a significant challenge, as much of it is landfilled or incinerated, with very low utilization of its chemical content. This results in a massive waste of resources and solid contamination of the environment [2].

Tomatoes are universally recognized as being among the most popular and widely consumed vegetables globally [3]. According to the World Tomato Processing Council (WTPC) report, more than 130 million tons of tomatoes are processed worldwide each year. Tomato fruits are highly perishable due to their high water content, which makes them susceptible substrates for the growth of microorganisms which results in subsequent spoilage by these microbes [4]. However, this large-scale processing generates significant waste; about 8 million tons, representing a waste of about 6.15% of processed tomatoes [5]. Additionally, a substantial portion of tomato production is unsuitable for consumption and thus discarded because it does not meet the desirable characteristics of color, ripeness, and health. Besides processing, several factors influence the quality of agricultural products, such as the level of fruit ripeness, farming practices, soil quality, and other variables like storage environment, which affect their nutritional value and represent an economic loss for producers [6,7]. China is the world’s leading producer of tomatoes, closely followed by India and Turkey. These leaders are joined by the United States and Egypt, forming a group of five key players that constitute around 70% of global tomato production [8].

They provide a diverse complement of essential organic compounds and exert a multifaceted influence that encompasses the modulation of pigmentation, delayed aging processes, lipid and blood pressure reduction, prostate health protection, and strengthening of the gastric and hepatic systems by reinforcing gastric and hepatic functions [9]. They also provide valuable bioactive ingredients for the body, such as lycopene, a fat-soluble isoprenoid with an extensive diversity of biological characteristics. Lycopene has interesting antioxidant properties that can potentially prevent the risk of various diseases, such as cancer, degenerative nerve diseases, cardiovascular diseases, and eye diseases [10,11].

In addition to the anticancer properties associated with lycopene, tomatoes also play an essential role in reducing insulin-like growth factor (IGF) levels in the blood, which may be advantageous in preventing various types of cancer. They also intervene in the cellular pathways involved in cell proliferation and tumor progression, contributing to their potential as a protective food against cancer [12].

Regular consumption of tomatoes, which are naturally rich in carotenoids, has been reported to provide crucial protection against various health disorders. This includes preventing vitamin A deficiencies and reducing the risk of chronic diseases. Tomatoes are also rich in other vital antioxidants, particularly lutein and zeaxanthin, which maintain good visual condition. These antioxidants help prevent eye deterioration caused by oxidative stress from free radicals, reducing the risk of developing macular degeneration. Therefore, integrating tomatoes into our daily diet can contribute significantly to maintaining optimal eye health and preventing chronic diseases that affect long-term quality of life [9,13].

Tomatoes are processed into tomato sauce, puree, ketchup, soup, and juice. They are even used in cosmetics, such as homemade face masks and serums, since they contain biotin and vitamin C. Tomatoes deserve attention due to their taste and gastronomic advantages, as well as the presence of biologically significant substances, including vitamins and minerals. Work is underway to create varieties of tomatoes rich in anthocyanins, such as “Sun Black” tomatoes [14]. However, 10% to 30% of tomato weight is transformed into waste during processing [15]. Tomato pomace, a by-product of tomato processing, usually consists of approximately 56.0–65.3% skin, a portion of the pulp, and 38.5–44.0% seeds [16]. The management and disposal of pomace represents significant environmental risks due to its unstable organic compounds and high enzymatic activity. The microbial decomposition of tomato waste puts human health at risk, so treating this waste is imperative and requires more significant investment by the government and the productive sector [17].

After tomato harvesting, several by-products or residues remain, such as the foliage and stems of the plant and tomatoes that do not meet quality standards for marketing [18]. Biomass residues generated by the tomato processing industry represent approximately 2% to 5% of the total production process, equivalent to hundreds of thousands of tons of waste annually. These compounds can trigger decomposition and fermentation processes, releasing pollutant gases such as methane, carbon dioxide, and leachates that contaminate ground and surface water with harmful chemicals [18,19]. It is common for tomatoes that do not meet quality standards to be discarded. Therefore, it is necessary to establish a system to recover nutrients from these wastes.

This systematic review presents an overview of the use of tomato waste to obtain lycopene, a compound with broad therapeutic potential in several types of diseases. Likewise, it seeks to contribute to advancing knowledge on the rational use of tomato waste in producing bioactive compounds with potential for cancer treatment. Understanding the mechanisms of action of lycopene and other tomato peel components can establish a solid basis for developing new, safer, and more effective therapeutic interventions.

## 2. Methodology

The PRISMA 2020 methodology was used, taking into account the eligibility criteria for data collection. The first searches used Boolean connectors linking the most relevant keywords (or search terms). For example, “Solanum lycopersicum”, “tomato peel”, and “lycopene” in PubMed, Google Scholar, and Scopus databases, making use of the descriptors of each term using the DeCS server (DeCS—Descriptors in Health Sciences (accessed on 15 March 2023) and similarity terms from the National Center for Biotechnology Information (Home—MeSH—NCBI (nih.gov)). “Limit to” and “Exclude” filters were applied. The search was limited to the English language by using the “LIMIT-TO (LANGUAGE, “English”)” filter, and all keywords that were not related to the central search topic were excluded. Then, for every result obtained, the titles and abstracts were reviewed to exclude results unrelated to the central theme and to refine the search [20].

**Figure 1:**
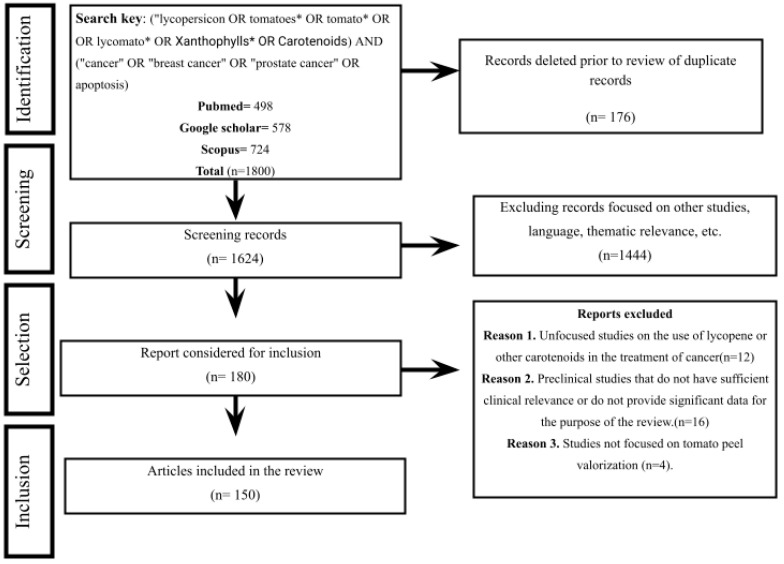
PRISMA 2020 methodology flow chart.

The reports obtained were selected according to the guidelines of the PRISMA 2020 methodology, which are summarized in Figure 1. The methodology consisted of an identification phase in which the number of records obtained in the central database (in this case, Scopus) and the number of records obtained from other sources or secondary databases (Google Scholar and PubMed) were reported. Then, a screening phase was applied in which duplicate citations were eliminated from the records obtained using the Mendeley reference manager, and the number of reports excluded was reported. Finally, the document inclusion stage was applied. In this stage, the number of reports included in the search is reported for complete analysis of their information.

Additionally, the PICO (patient, intervention, comparison, outcome) methodology was used to construct the search key with which we tried to obtain information related to the physicochemical characteristics of tomato peel for producing biofertilizers and lycopene for therapeutic applications [21,22].

## 3. Tomato

Tomato (*Lycopersicon esculantum* Mill.) is a vegetable in the Solanaceae family of flowering plants, encompassing over 3000 species. Notably, the lone domesticated species within the Lycopersicon section comprises 13 species or subspecies (Table 1) [23,24].

Tomatoes, the second most consumed vegetable worldwide, are appreciated for their fresh and healthy qualities and play a vital role in human nutrition [26]. This importance is derived from their rich composition of functional compounds, which make them a good source of antioxidants, including lycopene, vitamin C, vitamin E, β-carotene (a precursor of vitamin A), and phenolic compounds such as flavonoids (Table 2) [27,28].

Vitamin C is crucial in collagen biosynthesis, an essential structural protein. This vitamin is an enzymatic cofactor in several critical steps of collagen synthesis and participates in protein metabolism [29]. Moreover, tomatoes contribute additional antioxidants like β-carotene, hydroxycinnamic acid, chlorogenic acid, homovanillic acid, and ferulic acid [12].

**Table 2:** Chemical and caloric composition of tomatoes.

Content	Unit	Ref.
Energy	(8351–15,375) J/s	[9,30]
Proteins	(5.4–17.71) g/100 g d.m.	[9]
Lipids	(3.22–23.44) g/100 g d.m.	[31]
Ash	(0.02–0.5) g/100 g d.m.	[32]
Carbohydrates	(3.9–5.92) g/100 g d.m.	[33]
Fiber	(1.2–11.44) g/100 g d.m.	[34]
pH	(4.68–3.38)	[35]
Glucose	(0.48–2.45) g/100 g d.m.	[36]
Sucrose	(0.02–0.05) g/100 g d.m.	[37]
Acidity	(0.07–0.48) g/100 g d.m.	[38]
Water content	(68.03–96.17) g/100 g d.m.	[39]
Lycopene	(410.6–445.2) mg/100 g d.m.	[40]
Vitamin C (ascorbic acid)	(255.7–39.4) mg/100 g d.m.	[41]
Vitamin E	(0.17–0.1) mg/100 g d.m.	[42]
β-carotene	(0.62–1.2) mg/100 g d.m.	[43]
Phenolic acids	0.07372 g/100 g d.m.	[44]

d.m. = dry matter.

Key factors that help characterize and identify tomato nutrients include energy content, acidity level, and concentration of reducing sugars such as sucrose, fructose, glucose, and others. As tomatoes grow, the soluble sugars and organic acids act as solutes in their cells, allowing them to attract and retain water for proper water balance. This balance is crucial for maintaining cell turgor and structure, ultimately impacting the tomato’s texture, flavor, and overall quality. These soluble sugars and organic acids, accounting for over 50% of their dry matter, are the primary osmoregulatory substances in tomatoes, significantly contributing to their osmotic balance and overall quality [26].

### 3.1. World Tomato Production

**Figure 2:**
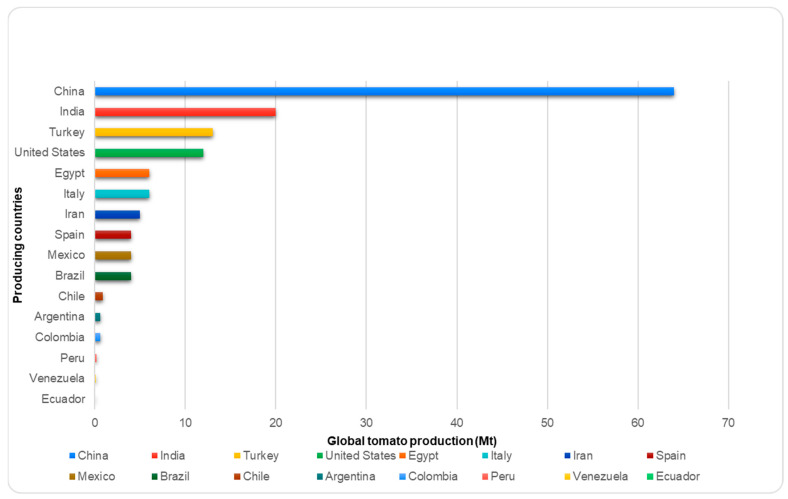
Central tomato-producing countries in the world.

Global tomato production is estimated at around 189 megatons (Mt) per year [45]. China is the world’s largest tomato producer, with an output of 56.4 Mt per year, representing 41.4% of global production (Figure 2). In 2020, China dedicated 1.1 Mha to tomato production [46]. India ranks second in production, followed by Turkey, the United States, Egypt, Italy, Iran, and finally, Spain, with an average of 4.7 megatons (Mt).

In 2021, tomato exports from China experienced a remarkable 24% increase compared to the previous year, driven by the rise in supply to 142 countries. This increase followed a sharp contraction during the COVID-19 pandemic. Additionally, there was a notable increase in crop yields and processed quantities [47]. In 2022, global tomato production reached almost 186.82 million tons. However, the global tomato market was projected to grow from USD 165.72 billion in 2022 to USD 178.65 billion in 2023 at a compound annual growth rate (CAGR) of 7.8%. The Russia–Ukraine war has negatively impacted expectations for global economic recovery after the COVID-19 pandemic [48]. Although projections for 2024 are not yet available, growth is expected to continue in line with the trends observed in previous years.

Tomato production thrives in several regions of Colombia, with the highest concentration in the department of Boyacá. Other significant tomato-producing areas include the departments of Cundinamarca, Norte de Santander, Valle del Cauca, Huila, Antioquia, Risaralda, and Caldas [49]. In Colombia, tomatoes are prominently featured as one of the primary vegetables, boasting an annual per capita consumption of 9.4 kg and a national production of approximately 0.6 megatons (Mt) [50].

### 3.2. Tomato Processing

The urgency to ensure a sufficient supply of fresh tomatoes for consumption or for processing other products often results in overproduction, leading to large volumes of waste. In addition, overproduction is a frequent tactic in the industry to compensate for possible losses during processing, including tomatoes discarded due to non-compliance with quality standards [51].

**Figure 3:**
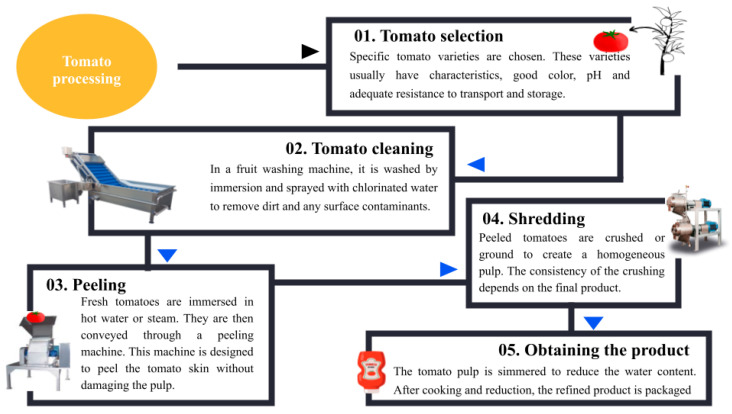
General scheme of tomato processing at the industrial level.

Tomatoes are commonly consumed as food or used to create various processed products. About 40 Mt of tomatoes are processed annually, making them the most processed vegetable by weight [52]. The vegetable samples undergo processing steps, including tomato sorting, washing, peeling, refining, and obtaining derived products (Figure 3) [53]. After harvesting, tomatoes are taken to the processing plant within hours. They undergo a meticulous selection process to determine their quality [54]. When assessing tomato quality, several key aspects are considered to ensure the fruit’s freshness, flavor, and overall appearance. First, the color of the fruit, indicative of ripeness, is evaluated. The soluble solids content, which includes sugars and other dissolved compounds, is measured. The pH level, representing acidity or alkalinity, is examined, as this can influence flavor perception and shelf life. Additional defects to be considered include worm damage caused by pests, mechanical damage resulting from handling or transportation, mold growth due to improper storage conditions, blemishes affecting the appearance of the fruit, and rot caused by microbial activity or other factors [55].

After quality evaluation, a critical step in the production process of tomato products is carried out: washing. Washing involves using water, often chlorinated, to remove contaminants on the tomato surface, such as soil, chemical residues, or microorganisms. Chlorine is used to disinfect the water, minimizing the presence of spores and other microorganisms that could compromise the quality of the final product.

Through a series of unit operations, tomatoes undergo heat treatment to break and loosen the skin of whole tomatoes. Following immersion in the peeling medium, typically hot water, the tomatoes undergo vacuum cooling before being transported to drag rollers. This process aids in the complete removal of the skin [56]. In the next step, the tomatoes are cut and crushed, then they can be processed using thermal methods. There are two methods for thermal processing of fresh tomatoes. The hot break process entails subjecting fresh tomatoes to elevated temperatures, in contrast to the cold break process, during the cutting phase. The primary distinction between these two methods is the apparent viscosity of the final product. The cold break process is generally carried out below 70 °C, while the hot break process is carried out at temperatures between 85 and 102 °C [57,58,59]. Finally, after crushing, the processed tomatoes undergo an additional process to remove the seeds and skin. This process may include using an extractor, pulper, or finisher, which helps separate unwanted components from the tomato puree. The juice is then extracted using a screw or paddle extractor, which allows a high-quality final product without impurities to be obtained. This step is crucial to ensure the purity and desired texture of the tomato juice [57].

### 3.3. Waste Production during Tomato Processing

The primary by-product of the tomato processing industry is tomato pomace, which is mainly comprised of peels and seeds. This waste accounts for around 3% of the fresh tomatoes processed, translating to approximately 0.8 million tons annually. A significant challenge in tomato pomace management is its seasonality. Tomato processing is concentrated over 2–3 months, producing large daily volumes of waste during this period. This concentrated production season creates significant challenges for effective waste management [60]. Tomato seed waste, a by-product of fruit processing, harbors many bioactive compounds with potential health benefits. Research using in vitro and in vivo models has revealed that extracts from these seeds have various promising activities, such as antiplatelet, antioxidant, anticancer, antimutagenic, antimicrobial, and neuroprotective activities [61]. This concentrated production season, in turn, creates significant challenges for effective waste management. Tomato waste residue, rich in antioxidants such as lycopene (510.6 mg/kg), β-carotene (95.6 mg/kg), and total phenols (1229.5 mg GAE/kg), has promising future applications. The initial moisture content of this by-product is around 66–67% (wet basis), and tomato peel (61.5%) and seeds (38.5%) make up the majority of its composition [62].

#### 3.3.1. Tomato Peel

Understanding the anatomy of the tomato peel is essential, as it houses valuable components necessary for health and nutrition. These include lycopene, dietary fiber, pectin, proteins, oil, and antioxidants, all contributing to the fruit’s nutritional value and potential health benefits [63]. Cutin is an essential component of tomato peel, constituting 45% to 80%. Cutin is an amorphous polyester consisting of long-chain fatty polyhydroxy acids of 16 or 18 carbons linked by cross-ester bonds, and the specified fatty acids dominate the structure of cutin [64]. The tomato-based food processing industry generates a considerable amount of waste. For example, from 10 kg of tomatoes, only 1 kg of pulp is obtained, which means that the remaining 9 kg is considered waste and not used. This waste, known as tomato peel, represents a secondary raw material that can be used to obtain bioactive compounds using various procedures [65].

Recently, research has focused on the relationship between the bioactive compounds in tomato pulp and peel and their impact on seed production and flower and fruit development. This approach identifies the most promising tomato varieties in terms of nutraceutical compounds under drought-stress conditions and optimal conditions [66] This knowledge is crucial for selecting tomato varieties with improved nutritional profiles and increased resistance to adverse environmental conditions.

#### 3.3.2. Chemical Composition of Tomato Peel

The composition of tomato peel is rich and varied, with approximately 10.5 g of protein, 5.9 g of ash, 4.0 g of oil, and 78.6 g of carbohydrates per 100 g of dry weight [67]. Additionally, tomato processing by-products, such as peels and seeds, are particularly notable for their content of beneficial compounds. For example, they are rich in polyphenols, with levels exceeding 1000 mg/kg, and in fibers, which account for around 50% by weight. They also contain proteins, ranging from 10 to 18% by weight, and carotenoids, such as β-carotene (approximately 95 mg/kg) and lycopene (around 500–800 mg/kg) [65].

Tomato peel comprises an outer epidermal layer covered by a thin cuticle formed mainly by a polyester cutin, which is 4 to 10 lumens thick, in addition to hemicellulose and pectins [68]. The epidermal layer is highly hydrophobic and crucial as the fruit’s first defense against desiccation [69]. It also contains many minerals such as K, Na, Ca, Mg, Zn, and Fe. Tomato peel also contains Mn (1.4 mg/100 g dry matter), Cu (1.1 mg/100 g dry matter), Cr (0.06 mg/100 g dry matter), Ni (0.66 mg/100 g dry matter), Se (0.01 mg/100 g dry matter), and Co (0.01 mg/100 g dry matter) [65].

#### 3.3.3. Carotenoids

**Figure 4:**
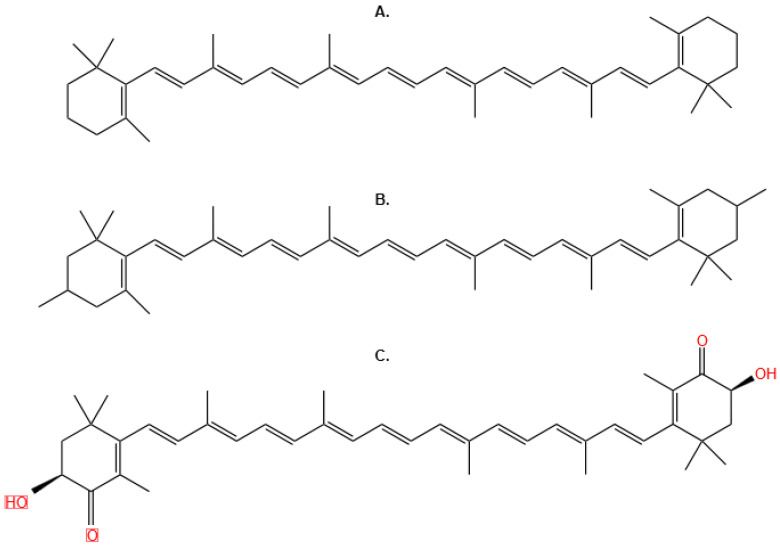
Examples of Carotenoids (**A**) β-carotene (**B**) lutein (**C**) zeaxanthin.

In tomatoes, carotenoid pigments are produced in the leaves, flowers, and fruits. In the leaves, these pigments protect against light, with lutein being the predominant carotenoid in this tissue [70]. The chemical structure of carotenoids exhibits a wide variability (Figure 4). Factors such as the arrangement of conjugated double bonds, geometrical changes due to isomerization, and the presence or absence of cyclic rings in the main polyene chain generate a diversity that comprises more than 600 carotenoid variants [71]. In addition, carotenoids are used to develop functional foods or as additives in food products to prolong their shelf life [72]. There is a growing interest in extracting carotenoids from tomato waste products. These pigments are valuable as natural food colorings and functional ingredients. However, traditional methods using organic solvents pose health and environmental risks because carotenoids are fat-soluble, so researchers have explored using vegetable oils for extraction. This approach could improve the functionality and heat resistance of the oils, making them more suitable for food applications [73].

Carotenoids are critical not only as essential nutrients for human health but also as precursors to the production of several vital phytohormones, such as abscisic acid (ABA) and strigolactones (SLs), which control plant growth and development [74].

The lipophilic nature of carotenoids complicates their direct integration into aqueous media, which has prompted the development of various vehicles such as emulsions, nanoemulsions, liposomes, hydrogel particles (a matrix of hydrophilic polymers that encapsulate solvent molecules), and solid–liquid particles (crystallized lipids dispersed in oil) [75]. Of these options, emulsions are widely employed in carotenoid formulations. Encapsulation techniques preserve carotenoids’ antioxidant activity, stability, and bioavailability, improving their functional performance by increasing their water dispersibility, solubility, and antioxidant capacity. As a result, carotenoids become more stable and effective [75,76,77].

#### 3.3.4. Carotenoid Extraction

Organic solvents are commonly used to extract carotenoids. The choice of solvents is critical and depends mainly on the polarity of the carotenoids. A combination of hexane, ethanol, and acetone is used to extract both polar and nonpolar carotenoids. In addition, accelerated solvent extraction has improved the extraction of tomato processing waste, such as the skin and seeds [78,79].

The principles of green extraction emphasize using methods that save energy, employ environmentally friendly solvents, use natural and renewable materials, and ensure the safe and high-quality extraction of bioactive compounds [80]. The widespread use of solvents in food processing has raised concerns about public health, safety, and environmental impacts. This is because some solvents are derived from petroleum and can produce harmful volatile compounds. Consequently, a concerted effort has been made to refine carotenoid extraction processes and identify alternatives to conventional solvents [81]. Thus, an innovative process using environmentally friendly solvents and eco-friendly techniques to avoid degradation of the target compound is of great interest [82]. Deep eutectic solvents (DESs) are emerging as a game changer in extraction procedures. These innovative solvents are formed by simply combining a hydrogen bond donor and acceptor, resulting in a mixture with a melting point lower than either individual component. This unique characteristic makes DESs highly tunable, allowing researchers to customize their properties for specific extraction needs [83].

#### 3.3.5. Lycopene

**Figure 5:**
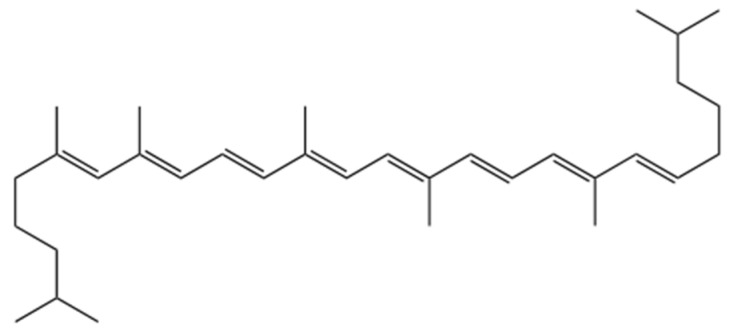
Chemical structure of lycopene.

Lycopene (ψ,ψ–carotene) is an acyclic tetraterpenoid hydrocarbon with a structure that includes 13 carbon–carbon double bonds, 11 of which are linearly conjugated (Figure 5) [72]. This arrangement of double bonds gives it a high conjugation degree, making lycopene one of the most potent antioxidants known; in fact, its antioxidant capacity is about twice that of β-carotene and ten times greater than that of tocopherol [84].

The primary biological function of lycopene lies in its ability to protect cellular DNA from oxidative stress. By acting as an antioxidant, lycopene helps prevent DNA damage that could otherwise lead to the development of chronic diseases. Its molecular structure, which consists of a long chain with multiple conjugated double bonds, gives it a remarkable ability to trap and neutralize reactive oxygen species (ROS). In fact, among more than 600 natural carotenoids, lycopene stands out as the most potent ROS scavenger [85]. In addition to its role as an antioxidant, lycopene has also been associated with several health benefits. It has been suggested that its consumption may help reduce the risk of cardiovascular diseases, certain types of cancer, and age-related eye diseases, among other potential benefits. These properties make lycopene a compound of interest in biomedical research and the food industry.

The term apoptosis is often used synonymously with programmed cell death. However, in a more specific sense, programmed cell death can refer to other forms of cell death that involve gene expression without necessarily meeting all the morphological criteria of apoptosis. When damaged cells do not follow the apoptosis process, they can become immortal and potentially develop into cancer cells. Recent research, including several in vitro studies with human prostate, ovarian, breast, stomach, and other cancer cell lines, has shown encouraging results regarding the potential of lycopene to induce apoptosis in these cells. This suggests that lycopene could be an essential chemotherapeutic agent [86,87]. Furthermore, lycopene has been shown to inhibit cell proliferation, induce cell cycle arrest at various phases, and enhance apoptosis in cancer cells. These findings indicate that lycopene can modulate the activity of cell cycle regulatory proteins, thus offering a possible mechanism for its anticancer effects [88].

#### 3.3.6. Lycopene Extraction from Tomato Peel

The increasing demand for lycopene for various uses in the food, pharmaceutical, and cosmetic industries has motivated scientists to seek alternative methods for its extraction from natural sources. The efficiency of lycopene recovery can vary due to several factors, such as the extraction method, the type of solvent used, and the location of lycopene in the cells. Depending on each study’s specific objectives, various techniques can be employed, such as those assisted by ultrasound or microwaves [89]. Several methods have been developed to extract lycopene from tomato skin, each with particularities and advantages (Table 3).

Maintaining precise environmental conditions ensures optimal lycopene extraction, handling, and analysis. To prevent light exposure that degrades lycopene, it is highly recommended to utilize light sources that emit golden, yellow, or red hues. Moreover, utilizing antioxidants such as butylated hydroxytoluene (BHT) in solvents can effectively control oxidation and isomerization reactions [90].

**Table 3:** The most common methods for lycopene extraction.

Technique	Principle of Operation	Operating Conditions	Lycopene Extraction	Environmental Considerations	Refs.
Supercritical fluid extraction with CO_2_ (SC-CO_2_)	Employing carbon dioxide within supercritical extraction methods implements the supercritical fluid extraction (SFE) technique. This separation method utilizes a solvent fluid in a supercritical state to conduct the extraction process.	Pressure, temperature, CO_2_ flow rate, and extraction time.	Lycopene extraction uses supercritical carbon dioxide (SC-CO_2_) as a solvent. This process takes advantage of the supercritical properties of CO_2_, acting as a highly efficient solvent to extract the lycopene-containing tomato oleoresin. SC-CO_2_ penetrates the plant material during extraction and selectively dissolves the lycopene from the tomato matrix.	Employs environmentally friendly methods, eliminating the need for organic solvents and reducing storage, disposal, and environmental risks.	[91,92,93]
Enzyme-assisted extraction (EAE)	This method entails employing enzymes to enhance the effectiveness and specificity of extraction procedures. Through collaborative action with the enzymes inherent in the matrix, EAE enables a more effective breakdown of cellular structures, thereby aiding the liberation of the desired compounds.	Optimal enzyme conditions of temperature, pH, and dosage; optimal time-temperature conditions; plant material such as particle size, water content, chemical composition, and solvent-to-solid ratio.	Enzyme-assisted extraction is used to obtain lycopene. Enzymes such as cellulases, pectinases, and glucanases, individually or in combination, hydrolyze the bonds present in plant cell wall polysaccharides.	Mild conditions, extract quality, higher extraction yields, and higher quality.	[94,95,96]
Microwave-assisted extraction (MAE)	A technique in which microwave radiation is utilized to warm solvents in contact with a sample, facilitating the extraction of analytes from the sample matrix into the solvent.	Solid-to-solvent ratio, extraction time, and microwave power for extraction yield.	The application is the optimization of lycopene extraction from tomato peels using the microwave-assisted extraction (MAE) technique. The main objective is to improve the extraction efficiency of lycopene, a carotenoid present in tomato peel, which can be used as a natural colorant or bioactive ingredient.	MAE exploits a small number of solvents, so it is considered a “green” technique. Moreover, heating occurs selectively with much less energy loss in the environment.	[97,98,99,100]
Optimized mixed-polarity solvent mixtures	This method is frequently utilized in the food and pharmaceutical sectors due to its efficacy in extracting lipophilic compounds such as lycopene.	Extraction temperature, type of solvent used, stirring time, sample volume, and filtration method.	It is based on using homogeneous mixtures of solvents that exhibit two distinct properties: (a) high affinity for lycopene and (b) ability to swell the plant material and thus improve solvent penetration.	By optimizing solvent combinations, it is feasible to reduce the total amount required to perform an extraction or separation, reducing natural resource use and waste production.	[101]
Ultrasonic-assisted extraction	The ultrasound-assisted extraction (UAE) technique is based on acoustic cavitation, which occurs due to the propagation of mechanical waves generated by alternating high- and low-pressure cycles, known as compressions and rarefactions.	Properties of the solvent involved in extraction, such as viscosity and surface tension, alongside environmental factors like temperature and pressure.	Recent studies suggest that ultrasonic extraction enhances the extraction speed and boosts the yield of lycopene by approximately 10%.	Allow for the practical, cost-efficient, and eco-friendly extraction of bioactive components from plant sources. These technologies provide a sustainable and efficient means of producing top-quality plant-based products and offer a substantial competitive edge to businesses in the field.	[43,102,103,104]

##### Supercritical Fluid Extraction with CO2 (SC-CO2)

**Figure 6:**
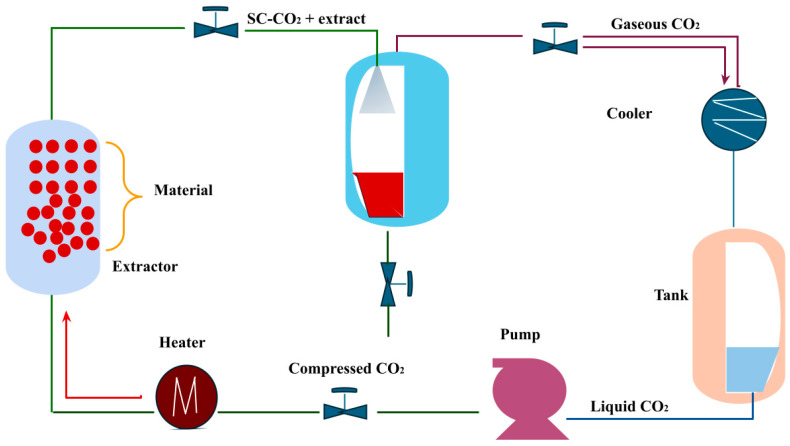
Scheme of lycopene extraction by supercritical fluids.

Supercritical carbon dioxide extraction technology (Figure 6) represents an environmentally friendly alternative to conventional organic solvents. This technology uses a supercritical fluid solvent for extraction, standing out as an innovative option for separating components [93,105,106].

Supercritical CO2 is the predominant solvent employed in SFE primarily because of its low critical temperature (31.1 °C) and non-toxic nature, enabling the extraction of thermolabile compounds. Additionally, SC-CO2 is non-flammable, easily accessible, and cost-efficient. Being in a gaseous state at ambient temperature and pressure, it can be extracted from extracts by expanding to atmospheric conditions without further processing [107].

For extracting lycopene from tomatoes, utilizing supercritical fluid extraction with CO2 (SC-CO2) is preferable to organic solvent extraction due to its inherent advantages. In a particular study, SC-CO2 was employed to extract lycopene from tomato peel-containing seeds [107]. Various extraction parameters were assessed to identify the optimal conditions for achieving a high lycopene yield. It was determined that the highest quantity of lycopene was extracted under 300 bar pressure, 60 °C temperature, a CO2 flow rate of 2 mL/min, and an extraction duration of 60 min. The lycopene content in the extract was similar to that obtained through conventional organic solvent extraction techniques. These findings indicate that SC-CO2 represents a viable and environmentally friendly approach for extracting lycopene from tomato peel-containing seeds [107].

##### Enzyme-Assisted Extraction (EAE)

**Figure 7:**
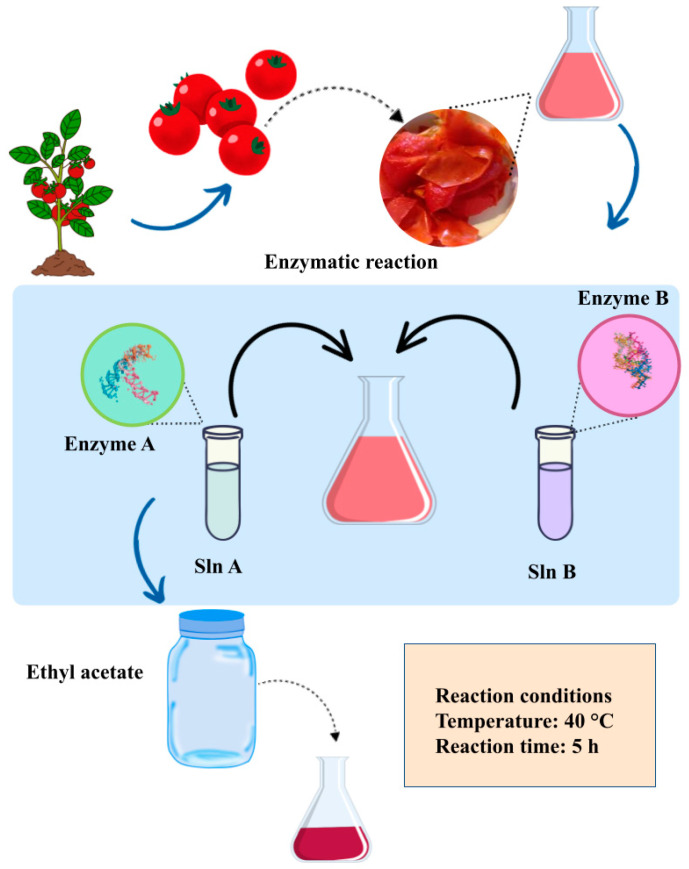
Scheme of lycopene enzyme-assisted extraction.

Enzyme-assisted extraction (EAE) (Figure 7) is a technique that harnesses the catalytic capabilities of particular enzymes to break down or alter cell walls, thus aiding in the liberation of intracellular compounds, primarily phenolic compounds, which are of interest [108]. The process entails the attachment of plant cells from biomass to the active sites of an enzyme, inducing a change in the enzyme’s conformation to accommodate the substrate at these active sites. Consequently, the active constituents within the cells are released into the extraction medium [109].

In research led by Gizem Catalkaya and Derya Kahveci, the optimization of lycopene extraction from tomato industrial waste was addressed [110]. The aim was to determine the most suitable solvent system and apply an enzymatic pretreatment to improve lycopene recovery. The results revealed that combining cellulolytic and pectinolytic enzymes followed by extraction with ethyl acetate produced lycopene oleoresins with an optimal concentration of phenolic compounds, enhanced antioxidant properties, and an exceptionally high red color intensity. The optimal extraction conditions were an enzymatic reaction temperature of 40 °C, an enzymatic reaction time of 5 h, an enzyme-to-substrate ratio of 0.2 mL/g, a solvent-to-substrate ratio of 5 mL/g, an extraction time of 1 h, and an enzyme-to-solvent ratio of 1. Under these conditions, a lycopene concentration of 11.5 mg per gram of oleoresin was achieved. This study demonstrated that the residues generated in tomato paste production can be used to obtain lycopene, a valuable component for both the food and nutraceutical industries, through the combination of enzymatic and solvent extraction [110].

##### Microwave-Assisted Extraction

Microwave-assisted extraction (MAE) involves the utilization of microwave radiation with a frequency close to 2.45 GHz (12 cm), leading to dielectric heating primarily caused by the absorption of energy by water and other polar compounds within the moist biomass or the specific sample [111]. The process of MAE for separating flavonoids can be delineated into three consecutive stages. Initially, flavonoids are liberated from the active sites of the sample matrix under elevated pressures and temperatures. Subsequently, the solvent is diffused through the sample matrix. Finally, the active flavonoids are released from the sample matrix into the solvent [112]. The MAE technique offers numerous advantages over alternative extraction methods, including reduced production costs, shorter processing times, decreased solvent requirements, and enhanced extraction efficiency with lower energy consumption and CO_2_ emissions [113].

A study focusing on microwave-assisted extraction (MAE) of lycopene from tomato peels aimed to optimize extraction conditions and assess yields of trans and cis isomers. Response surface methodology was employed to fine-tune the lycopene extraction, considering factors such as solvent ratio, solid–liquid ratio, microwave power, and energy input. The findings revealed that solvent ratio and microwave power notably influenced the lycopene extraction yield, with MAE proving more effective than conventional extraction methods. However, the obtained yields fell short of expectations, likely due to preheating of the tomato peels. Additionally, MAE induced more pronounced structural alterations in tomato peels, hinting at a potential reduction in physical barriers to extraction. These findings offer valuable insights for refining other extraction techniques to retrieve cis or trans isomers of lycopene, depending on the specific application requirements [100].

##### Optimized Mixed-Polarity Solvent Mixtures

One of these approaches relies on utilizing homogeneous solvent mixtures. This method has wide applications in the food and pharmaceutical sectors due to its ability to efficiently extract lipophilic compounds like lycopene. Since lycopene is a non-polar compound, the ideal extraction solvent should preferably be non-polar or slightly polar, with a low boiling point, to remove it via evaporation. These mixtures typically consist of organic solvents such as hexane, ethyl acetate, or chloroform, known for their affinity towards lipophilic compounds and efficient dissolution capabilities. Conversely, an effective swelling agent should possess relatively high polarity. Thus, efforts have focused on blended solvents that amalgamate these polarity attributes, aiming to optimize the extraction process efficiency [101].

##### Ultrasonic-Assisted Extraction

**Figure 8:**
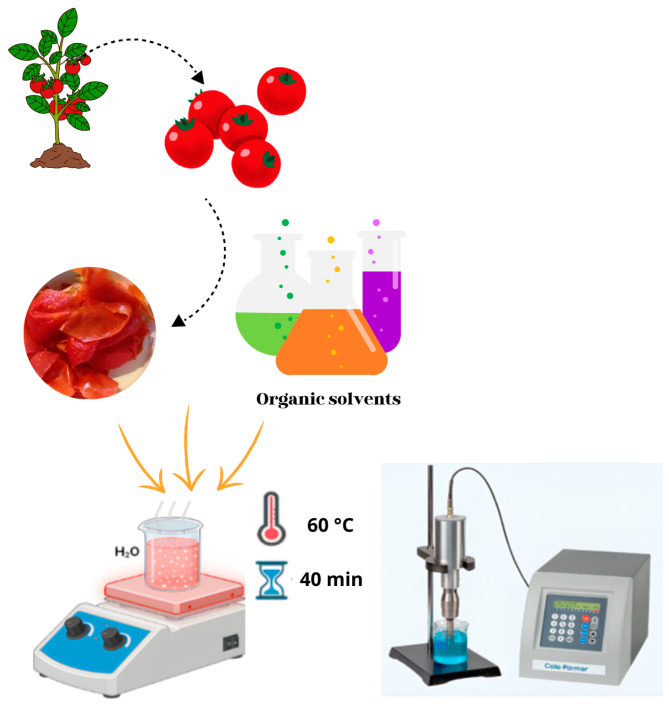
Scheme of lycopene ultrasonic-assisted extraction.

Ultrasonic-assisted extraction (Figure 8), heralded as an innovative and promising technique with a myriad of applications across the chemical, pharmaceutical, cosmetic, and food sectors in the 21st century, harnesses sound waves at approximately 20 kHz. These waves, generated by converting electrical energy into mechanical energy, propagate through gases and liquids [114,115]. Ultrasound-assisted extraction enables rapid acquisition of bioactive ingredients and is achieved at low temperatures, with reduced energy and solvent consumption.

Being a non-thermal extraction method, it effectively preserves the functionality of bioactive compounds. However, various process variables must be tailored individually for each feedstock, including frequency, power, duty cycle, temperature, time, solvent type, and liquid-to-solid ratio [116].

Ultrasound-assisted extraction (UAE) of lycopene from tomato paste processing residues was investigated and compared with the conventional organic solvent extraction method (COSE). Lycopene extraction yields were evaluated using different solid-to-solvent ratios, temperatures, and ultrasonic powers. It was determined that, for COSE, the most efficient extraction was achieved with a solid-to-solvent ratio of 50:1 at 60 °C for 40 min, while for UAE, it was achieved with a ratio of 35:1, and an ultrasonic power of 90 W for 30 min. It was observed that UAE required less time, a lower temperature, and less solvent for lycopene extraction than COSE. UAE proved more efficient than COSE, achieving higher lycopene yields in a shorter time and at lower temperatures. In addition, ultrasonic application was observed to accelerate the extraction rate and increase the yield by approximately 10%. Extraction time was determined to be the most critical factor in UAE, as an increase in lycopene yield was observed with time, but no significant changes were observed after 30 min. Optimization of UAE is crucial, as incorrect conditions could lead to degradation of the desired component due to the excess heat and pressure generated by acoustic cavitation [117].

#### 3.3.7. Antioxidant Activity of Lycopene

Oxidative stress refers to imbalances between pro-oxidant and antioxidant processes that result in cellular oxidation. This phenomenon originates from an imbalance between the production and accumulation of reactive oxygen species (ROS) in cells and tissues and the capacity of the biological system to detoxify these reactive substances effectively [118]. This imbalance can cause damage to cellular DNA, proteins, and lipids, which in turn can be linked to the development of various chronic diseases [119]. Oxidative stress is recognized as one of the factors responsible for an increased risk of cancer [120]. On the other hand, lycopene has been shown to reduce oxidative damage by increasing the levels and activity of antioxidant enzymes, such as glutathione (GSH), glutathione S-transferases (GST), and glutathione peroxidase (GPx), as well as superoxide dismutase (SOD) and catalase (CAT). These enzymes are essential for neutralizing free radicals and protecting cells from oxidative stress [121].

Due to its polyene structure and numerous conjugated double bonds, lycopene exhibits high reactivity towards oxygen and free radicals. It is recognized as the most effective antioxidant in neutralizing singlet oxygen, surpassing the other 600 natural carotenoids. Each lycopene molecule can neutralize approximately 1000 oxygen molecules. The presence of conjugated double bonds makes it susceptible to oxidation reactions, making it a potent antioxidant capable of neutralizing singlet oxygen and scavenging free radicals. This protects it against oxidative stress and related cellular damage [122].

Lycopene is crucial for cellular protection against oxidative stress. This fat-soluble compound modifies reactive oxygen species (ROS) through three main mechanisms. First, lycopene forms adducts with free radicals through radical addition, stabilizing them and reducing their harmful effect. Then, through electron transfer, lycopene can neutralize free radicals by donating electrons, converting them into less reactive forms. Finally, lycopene reacts with free radicals as a hydrogen donor through allylic hydrogen abstraction, neutralizing them and preventing oxidative damage. These reactions can co-occur and are influenced by factors such as the types of free radicals, the molecular structure of lycopene, and its location in the cell membrane. Lycopene has been observed to exhibit higher antioxidant activity in biological systems, such as cells and tissues, which highlights its importance in cellular protection against oxidative stress and related damage. Studies have shown that an adequate intake of lycopene through diet may contribute to the prevention of chronic diseases such as cardiovascular conditions, eye-related issues, inflammatory diseases, and various types of cancer [123,124]. Lycopene can stop lipid peroxidation and protect DNA against damage by activating enzymes in the cellular antioxidant systems and promoting the expression of components responsible for the antioxidant action [125].

#### 3.3.8. Anticancer Activity of Lycopene

**Figure 9:**
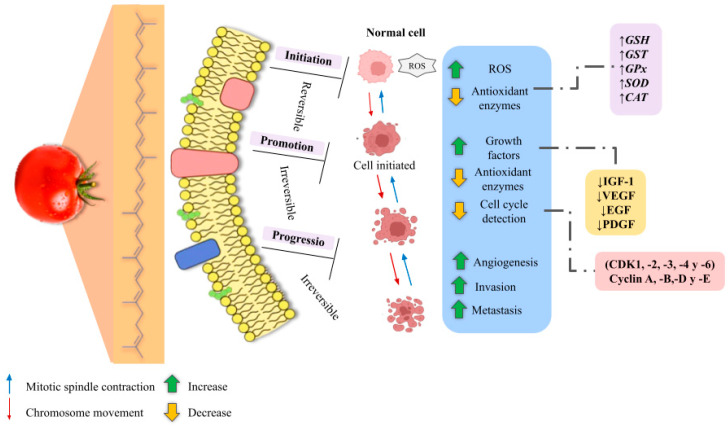
Stages in which lycopene intervenes in the carcinogenic process. Adapted from [129,130].

Recent research has reported on the direct anticancer properties of lycopene, which include the inhibition of growth factor signaling, cell cycle progression, and cell survival. This is achieved by modulating intracellular signaling pathways in tissues such as the endometrium, lung, colon, prostate, and breast cancer cells [126]. Figure 9 shows the stages of lycopene intervention in the carcinogenic process.

Cancer development is a multi-step process that includes initiation, promotion, and progression. The initiation phase of carcinogenesis, a complex process leading to cancer development, generally involves the introduction of carcinogens or exposure to radiation in previously healthy cells. This initial step poses a significant threat to cellular integrity and can lead to the accumulation of genetic mutations and oxidative damage. Lycopene, a natural pigment found abundantly in tomatoes and other fruits, emerges as a potential combat to this cancer initiation. Lycopene and its metabolites block this initial step by effectively neutralizing reactive oxygen species (ROS), stimulating detoxification mechanisms, and activating antioxidant enzyme systems. These actions collectively safeguard cells from the detrimental effects induced by carcinogenic initiators [127].

Furthermore, lycopene can impede tumor promotion and progression by regulating the essential signaling pathways activated by tumor promoters, inflammatory cytokines, and growth factors. By modulating these pathways, lycopene exerts a protective influence, thus contributing to the prevention and control of carcinogenesis [128,129]. The multifaceted actions of lycopene significantly contribute to the prevention and control of carcinogenesis. By neutralizing ROS, stimulating detoxification mechanisms, activating antioxidant defenses, and modulating critical signaling pathways, lycopene emerges as a promising natural compound for reinforcing cellular resistance against cancer initiation and progression [130].

Dysregulation of the cell cycle is a crucial characteristic of cancer cells, which often lose the ability to regulate their proliferation rate effectively. One critical step in the cell cycle that is frequently disrupted in cancer is the progression of cells through the first gap phase (G1). This dysregulation leads to uncontrolled cell division and contributes to the rapid growth of tumors [129].

Lycopene has been suggested to hurt cancer cells, or their development, by modulating cell cycle progression and proliferation. It has also been suggested that lycopene exerts an inhibitory effect on DNA synthesis while activating the upregulation of gap junction proteins and reducing local androgen signaling. These actions impact IGF-1 signaling, antioxidant activity, and the induction of apoptotic cell death. These findings suggest that carotenoids, such as lycopene, could offer a promising avenue for disease prevention. Their beneficial effects extend beyond simple antioxidant action, encompassing genomic and non-genomic aspects that can influence cellular health [131].

Lycopene has been shown to have antiproliferative, pro-apoptotic, and genotoxic effects on HT-29 colon cancer cells, and studies have shown that lycopene has the potential to be a promising therapeutic agent for colon cancer. The results indicate that lycopene significantly affects cell growth inhibition, apoptosis induction, and genotoxic damage in HT-29 colon cancer cells [132]. A second study focused on patients undergoing radiotherapy for breast cancer, who consumed 160 g of tomato juice daily for six months. While some studies use tomato juice, it is essential to acknowledge the limitations of this approach as it does not isolate the specific effects of lycopene. Prioritizing studies with isolated lycopene or exploring the potential synergistic effects in tomato juice would strengthen the evidence base. A significant increase in serum lycopene levels was observed, from approximately ~0.3 to 0.8 µmol/L from the end of the radiotherapy period to the end of the tomato juice consumption period. This increase suggests that tomato juice may be an effective source of lycopene, an antioxidant that could benefit breast cancer patients undergoing radiation therapy [133].

Another study, with a diverse group of participants including 40 men and 31 postmenopausal women with a personal history of colorectal adenoma, a family history of colorectal cancer, or both combined, was conducted to investigate the effects of daily tomato juice consumption as a supplement. Over 8 weeks, the participants received a daily dose of 30 mg of lycopene via tomato juice. The study’s results revealed significant changes in the levels of specific serum proteins associated with colorectal cancer risk. In particular, an increase in the concentration of insulin-like growth factor binding protein-1 (IGFBP-1) was observed in women after lycopene supplementation. This increase, which is associated with beneficial effects on cell growth regulation and apoptosis, suggests a possible protective effect of lycopene in preventing colorectal cancer in postmenopausal women [51].

#### 3.3.9. Lycopene Contribution to Cancer Treatment

While lycopene does not possess provitamin A activity, its structure contains 11 conjugated double bonds, twice as many as β-carotene. This unique configuration enhances its ability to quench singlet oxygen, a reactive oxygen species implicated in the initiation of carcinogenesis [134]. It also modulates genetic functions, carcinogen-metabolizing enzymes, apoptosis, and immune function (Table 4) [135]. Consequently, lycopene plays a crucial protective role against these harmful reactions, thus contributing to its potential as a cancer-preventive compound.

### 3.4. Other Uses of Tomato Peel

Tomato peel has gained attention due to its potential applications in various fields. Research has shown that tomato peel can be used in agricultural practices, such as improving plant cultivation and developing new tomato varieties [145]. Additionally, studies have focused on the genetic parameters and agronomic performance of specific tomato peel varieties, indicating the potential to improve the quality and yield of these crops [146]. Likewise, the extraction and processing of tomato peel seeds has been explored, demonstrating their potential for the use and the benefits of the seeds [147].

**Figure 10:**
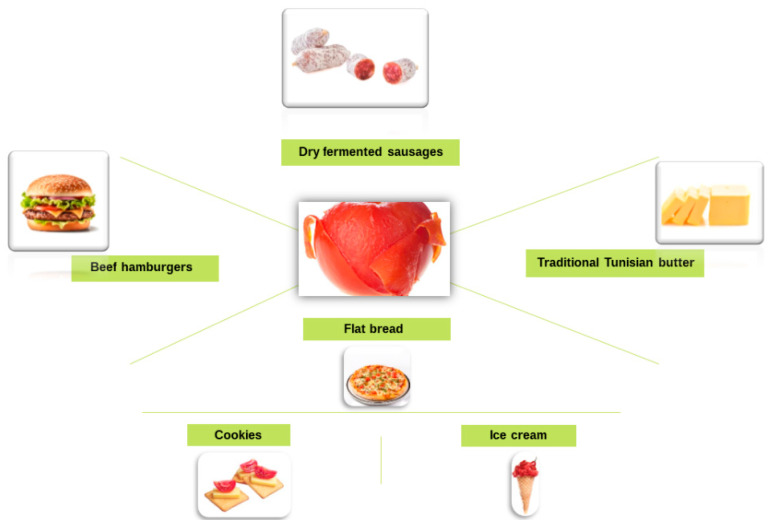
Different applications of tomato peel and extracts.

The application of tomato peel extends beyond agriculture, and research highlights its potential in food production (Figure 10). For example, the addition of dehydrated tomato peel and seeds has been studied to analyze their impact on the antioxidant capacity of tomato paste, indicating its potential applications in the food industry [148]. Additionally, the use of tomato peel as a substrate in crop rotation has been investigated, showing its potential in sustainable agricultural practices [149]. In conclusion, research on the uses of tomato peel covers various applications, from agricultural improvements to food production and sustainable practices. The results suggest that tomato peel has significant potential for practical and beneficial uses. The lycopene extract derived from tomato peel is also intended to be used as a food coloring. This extract is also used in the food industry as a food or dietary supplement in products that seek to obtain a specific value through lycopene [150]. It has been shown that enzymatic pretreatment of tomato peel with pectinolytic enzymes and surfactant-assisted extraction significantly increased lycopene recovery. Moreover, this eco-friendly process eliminates the need for organic solvents to extract lipophilic lycopene, ensuring its direct application in the food and cosmetic industries [151].

Innovative methods are being explored to enhance the nutritional value of meat products, particularly by incorporating dried tomato peels. When added to dry fermented sausages, these peels improve lycopene content and reduce lipid oxidation during storage, enhancing product acceptability. Similarly, adding tomato powder to frankfurters as a natural colorant and functional ingredient lowers pH and microbial activity, potentially extending shelf life while reducing nitrite levels. Moreover, ground beef enriched with dried tomato peels presents a healthier option due to increased lycopene and fiber content derived from the by-products of the tomato processing industry. These approaches offer opportunities to improve meat products’ nutritional profile and consumer appeal [151].

In Tunisian butter, using tomato extract as a natural antioxidant has shown promising results for shelf-life extension, with an optimum efficacy observed at a concentration of 400 mg/kg. The health benefits of the Mediterranean diet are well known, mainly due to the inclusion of tomatoes and olive oil. Co-milling olives with thawed or freeze-dried tomato by-products releases carotenoids, enriching the final product with lycopene without needing solvents or chemicals. Several functional food products have been enriched with industrial tomato by-products, presenting improved characteristics such as higher lycopene content, delayed oxidation, better sensory properties, and higher levels of dietary fiber and antioxidants [152].

In addition, low-quality edible oils have been valorized using tomato derivatives, particularly by fortifying refined olive oils with tomato peel to improve carotenoid content and serve as a natural stabilizer, replacing synthetic preservatives. These approaches offer sustainable alternatives to take advantage of tomato agro-industrial wastes while enhancing food products’ nutritional value and quality.

## 4. Conclusions

This review explores methods to extract lycopene and other beneficial carotenoids from tomato skin. Studies highlight their potential in cancer prevention and treatment due to their antioxidant, anti-inflammatory, and cell death-inducing properties.

Utilizing tomato peels, a common agricultural waste, reduces waste and promotes the use of natural antioxidants. Overall, this approach offers a promising strategy to address human health and environmental challenges by harnessing the power of tomato peel bioactives. This review also highlights the importance of adopting holistic approaches that address human health benefits, such as the anticancer properties of lycopene, and opportunities to improve environmental sustainability by utilizing organic waste in agricultural production.

## Figures and Tables

**Table 1:** Taxonomy of tomato. Adapted from [25].

Taxonomy	
Division	Angiosperms
Class	Magnoliopsida
Order	Solanales
Family	Solanaceae
Genus	*Solanum*
Species	*Solanum lycopersicum*

**Table 4:** Different cancer treatments based on lycopene.

Type of Cancer	Function of Lycopene	Administration	Studies	Type of Study	Refs.
Prostate cancer	Lycopene inhibits DNA synthesis, which could significantly decrease the proliferation and growth of cancer cells in primary epithelial prostate cancer.	Lycopene could be used as a therapeutic adjunct in patients with prostate cancer to improve apoptosis and prevent the progression of cancer cells.	Lycopene has demonstrated efficacy in treating locally advanced prostate cancer, reducing mortality in high-risk men, and slowing the progression of the disease.	In vitro	[134,136,137]
Breast cancer	Lycopene decreased cell growth, induced cell cycle arrest, and caused changes in mitochondrial membranes and DNA fragmentation. It showed no hemolytic activity and had low toxicity against peritoneal macrophages.	Supplementation with lycopene complexes and other antioxidants reduces skin toxicity during radical radiation therapy.	In an animal model, lycopene supplementation and other antioxidants demonstrated the potential to reduce skin toxicity during radical radiotherapy treatment for breast cancer.	In vitro	[134,138]
Lung cancer	Inhibits induced pulmonary toxicity by preventing inflammation and macrophage infiltration.	Adjuvant therapy.	Lycopene has been studied in laboratory and animal models of lung cancer cells. It shows cell growth-inhibiting properties and promotes apoptosis.	In vitro	[139,140]
Ovarian cancer	The consumption of lycopene through diet has been associated with a lower risk of ovarian cancer, indicating its potential as a preventive agent against ovarian carcinogenesis.	Lycopene, administered orally as a preventive measure, significantly reduced intraperitoneal metastatic burden and, when given as a treatment, significantly reduced the tumor burden of ovarian cancer.	Lycopene intake has decreased the occurrence and size of ovarian tumors in laying hens. This effect is attributed to its antioxidant and anti-inflammatory properties, which regulate signaling pathways in ovarian cells.	In vitro	[141,142]
Stomach cancer	Treatment with lycopene suppresses the proliferation of gastric cancer cells by inducing cell cycle arrest in the G0–G1 phase. Moreover, lycopene prevents the upregulation of p53 expression in gastric mucosa exposed to cigarette smoke.	The administration of lycopene at doses of 50, 100, or 150 mg/kg of body weight led to an anticipated increase in antioxidant enzymes (SOD, CAT, GSH-Px). It also increased cytokine levels (IL-2, IL-4, IL-10, TNF-α) and antibodies (IgG, IgA, IgM).	Lycopene intake protects against stomach cancer, regardless of *Helicobacter pylori*. Its beneficial effect in animal models of gastric and esophageal cancer lies in modulating the proliferation and apoptosis of tumor cells induced by carcinogens.	In vitro	[143,144]

## Data Availability

Data regarding the literature review will be made available through a request to the corresponding author.
